# Cannabinoid-Induced Stereoselective Inhibition of R-S-Oxazepam Glucuronidation: Cannabinoid–Oxazepam Drug Interactions

**DOI:** 10.3390/pharmaceutics16020243

**Published:** 2024-02-07

**Authors:** Keti Bardhi, Shelby Coates, Gang Chen, Philip Lazarus

**Affiliations:** Department of Pharmaceutical Sciences, College of Pharmacy and Pharmaceutical Sciences, Washington State University, 412 E. Spokane Falls Blvd, Spokane, WA 99202, USA

**Keywords:** cannabinoids, UGT, oxazepam, drug–drug interaction, cannabis, benzodiazepines, THC, CBD

## Abstract

Benzodiazepines (BZDs) such as oxazepam are commonly prescribed depressant drugs known for their anxiolytic, hypnotic, muscle relaxant, and anticonvulsant effects and are frequently used in conjunction with other illicit drugs including cannabis. Oxazepam is metabolized in an enantiomeric-specific manner by glucuronidation, with S-oxazepam metabolized primarily by UGT2B15 and R-oxazepam glucuronidation mediated by both UGT 1A9 and 2B7. The goal of the present study was to evaluate the potential inhibitory effects of major cannabinoids, Δ^9^-tetrahydrocannabinol (THC) and cannabidiol (CBD), and major THC metabolites, 11-hydroxy-Δ^9^-tetrahydrocannabinol (11-OH-THC) and 11-nor-9-carboxy-Δ^9^-tetrahydrocannabinol (11-COOH-THC), on the UGT-mediated metabolism of R- and S-oxazepam. The cannabinoids and metabolites were screened as inhibitors of R- and S-oxazepam glucuronidation in microsomes isolated from HEK293 cells overexpressing individual UGT enzymes (rUGTs). The IC_50_ values were determined in human liver microsomes (HLM), human kidney microsomes (HKM), and rUGTs and utilized to estimate the nonspecific, binding-corrected K_i_ (K_i,u_) values and predict the area under the concentration–time curve ratio (AUCR). The estimated K_i,u_ values observed in HLM for S- and R-oxazepam glucuronidation by CBD, 11-OH-THC, and THC were in the micromolar range (0.82 to 3.7 µM), with the K_i,u_ values observed for R-oxazepam glucuronidation approximately 2- to 5-fold lower as compared to those observed for S-oxazepam glucuronidation. The mechanistic static modeling predicted a potential clinically significant interaction between oral THC and CBD with oxazepam, with the AUCR values ranging from 1.25 to 3.45. These data suggest a pharmacokinetic drug–drug interaction when major cannabinoids like CBD or THC and oxazepam are concurrently administered.

## 1. Introduction

**Figure 1:**
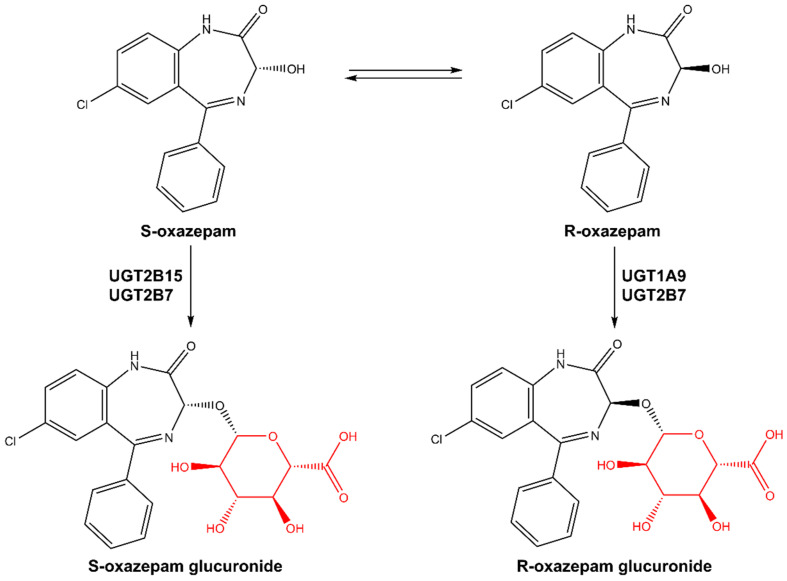
Metabolic pathways of R,S-oxazepam. Glucuronide motifs are highlighted in red.

**Figure 2:**
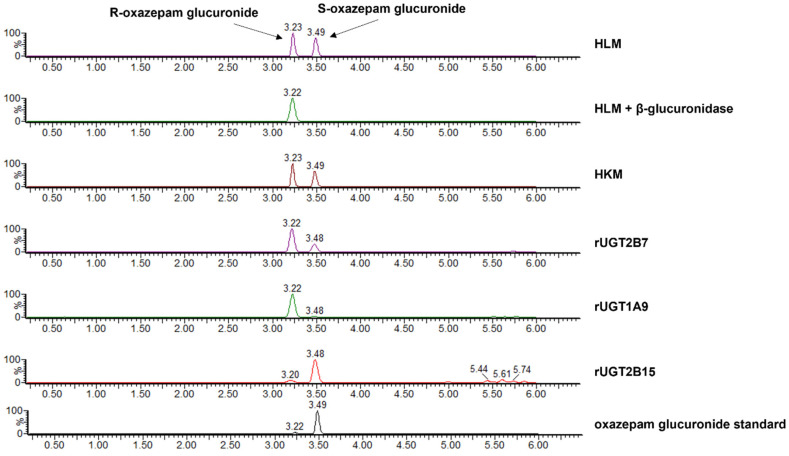
Chromatograms of R-oxazepam glucuronide (R) and S-oxazepam glucuronide (S) formed in assays containing R,S-oxazepam and HLM, HLM + β-glucuronidase, HKM, or microsomes from rUGT2B7, rUGT1A9, and rUGT2B15. The oxazepam glucuronide standard is shown in the bottom panel. The HLM + β-glucuronidase panel shows the chromatogram of oxazepam glucuronides present after HLM oxazepam glucuronide formation reactions are treated with β-glucuronidase for 10 min at 37 °C.

Oxazepam is a short-to-intermediate-acting 1,4-benzodiazepine (BZD) and is an active metabolite of several BZDs including diazepam, ketazolam, temazepam, and chlordiazepoxide [1,2]. Oxazepam is eliminated in humans primarily via glucuronidation of the 3-hydroxy group by multiple uridine diphosphate glucuronosyltransferase (UGT) isoforms [2,3,4] followed by urinary excretion, and has been used as an in vivo probe of drug glucuronidation in humans [1,2,5,6]. It exists as a pair of enantiomers (R- and S-) because of the chiral center at the 3-carbon position and is marketed as a racemate [1,5,7]. Several studies have investigated the UGT isoforms involved in the metabolism of R- and S-oxazepam [2,3], reporting that S-oxazepam is metabolized primarily by UGT2B15 with a minor contribution of UGT2B7, whereas R-oxazepam glucuronidation is catalyzed by both UGT 1A9 and 2B7 (see Figure 1; [2]). In vitro enzyme kinetic studies have shown that there is a higher clearance of S-oxazepam compared to R-oxazepam in the human liver [2,8]. These in vitro findings are consistent with the preferred glucuronidation of S-oxazepam in vivo, with S-/R- oxazepam glucuronide ratios in the plasma and urine of 3.5 and 3.9, respectively [2,8].

However, BZDs can be highly addictive, causing side effects and leading to dependence in many patients, occasionally indicating the need for additional treatments [9,10]. Cannabis has been used for many years to alleviate a variety of conditions [11,12]. Although prior research has investigated the interaction of BZDs with other medicines (opioids, antiepileptics, etc.) [13,14,15,16], the interaction with cannabis has received less attention, and the exact mechanism by which this drug–drug interaction (DDI) may occur is still unknown. As a result, combining cannabis with BZDs like oxazepam may cause complex DDIs, altering oxazepam clearance and resulting in prolonged sedation.

Cannabis is one of the most widely used illicit psychoactive substances both globally and in the United States and contains more than 150 different cannabinoids that produce a wide range of effects [17,18,19]. The prevalence of cannabis use among U.S. adults is anticipated to rise to approximately 46 million by 2025 [20]. Most cannabis use is recreational; however, cannabis and cannabis-derived substances are also used for medicinal and health purposes. The two most well-known and studied cannabis constituents are Δ^9^- tetrahydrocannabinol (THC) and cannabidiol (CBD). THC is the main psychoactive component of cannabis responsible for the known “high effect” and has antiemetic, analgesic, antispasmodic, and appetite-stimulating properties [21,22,23,24]. CBD induces either no or a weak euphoric response, binding to cannabinoid receptor (CB) 1 and CB2 in the brain with a much lower affinity than THC, but exhibits anxiolytic, muscle relaxant, anticonvulsant, and anti-inflammatory properties [13,25,26,27,28,29]. THC and CBD undergo hydroxylation or oxidation by cytochrome P450 (CYP450) enzymes, followed by glucuronidation via UGT enzymes, and are then excreted in the urine, bile, or feces [30,31,32,33,34]. Two major circulating THC metabolites found in the plasma of cannabis users include 11-hydroxy-Δ^9^-tetrahydrocannabinol (11-OH-THC) and 11-nor-9-carboxy-Δ^9^-tetrahydrocannabinol (11-COOH-THC). Like THC, 11-OH-THC is considered an active cannabinoid with psychotropic effects, as it interacts with cannabinoid receptors in the brain (both CB1 and CB2) [28,35]. The inactive metabolite 11-COOH-THC is derived from 11-OH-THC, peaking more slowly than 11-OH-THC in the blood of cannabis users but persisting in the blood at high and stable levels for a much longer time [36]. CBD has a similar pharmacokinetic profile to THC, with it metabolized to an active metabolite, 7-hydroxy-CBD (7-OH-CBD), and subsequently to 7-carboxy-CBD (CBD-COOH), which is the most abundant CBD metabolite in plasma [34,37].

Several studies have been conducted to evaluate the effects of major cannabinoids and their major circulating metabolites on drug-metabolizing enzymes (DMEs), including CYPs, UGTs, and carboxylesterases [20,38,39,40,41]. Recent studies have shown that THC, CBD, and their metabolites were found to inhibit the UGTs 1A9 and 2B7 [40], two of the UGTs important in oxazepam metabolism. The coadministration of the BZDs midazolam or clobazam with oral CBD resulted in the increased exposure of their active metabolites, 1-hydroxymidazolam and N-desmethylclobazam, respectively, due to inhibition of UGT or CYP enzymes [42,43,44]. Therefore, pharmacokinetic interactions between cannabinoids and BZDs like oxazepam could potentially result in adverse events, variability in clinical efficacy, or altered drug exposure. The goal of the present study was to evaluate the inhibitory potential of cannabinoids and their major metabolites on the UGT-mediated metabolism of R-and S-oxazepam and identify potential DDIs based on in vitro–in vivo extrapolation (IVIVE) modeling.

## 2. Materials and Methods

### 2.1. Chemicals and Reagents

Oxazepam, R,S-oxazepam glucuronide, ketoconazole, β-glucuronidase, and bovine serum albumin (BSA) were obtained from Sigma-Aldrich (St. Louis, MO, USA). Cannabinoids and their metabolites (THC, 11-OH-THC, THC-COOH, and CBD) were purchased from Cayman Chemicals (Ann Arbor, MI, USA) or Sigma-Aldrich after obtaining the appropriate Drug Enforcement Administration (DEA) licenses (state and federal). UDP-glucuronic acid (UDPGA) was purchased from Cayman Chemicals (Ann Arbor, MI, USA). Ultra-low binding microcentrifuge tubes, alamethicin, and magnesium chloride (MgCl_2_) were purchased from VWR (Radnor, PA, USA). Sekisui Xenotech, LLC (Lenexa, KS, USA) supplied pooled human liver microsomes (HLM; *n* = 50 subjects, mixed gender) and human kidney microsomes (HKM; *n* = 8 subjects, mixed gender). Dulbecco’s modified Eagle’s medium (DMEM), Dulbecco’s phosphate-buffered saline (DPBS), and fetal bovine serum (FBS) were all purchased from Gibco (Grand Island, NY, USA), while geneticin (G418) was purchased from Seradigm (Radnor, PA, USA). BCA protein assays were purchased from Pierce (Rockford, IL, USA). LC–MS-grade methanol, acetonitrile, and ammonium formate were obtained from Thermo Fisher Scientific (Waltham, MA, USA). Acquity UHPLC BEH C18 columns (1.7 µm 2.1 × 100 mm) were purchased from Waters (Milford, MA, USA).

### 2.2. UGT-Overexpressing HEK293 Cell Lines (rUGT) and Microsomal Preparation

Human embryonic kidney cells (HEK293) stably overexpressing the single UGTs 1A9, 2B7, and 2B15 have been previously established and described [45,46]. Cells were grown in DMEM supplemented with 350 μg/mL of geneticin and 10% FBS and harvested in PBS. Microsomal fractions of UGT-overexpressing cell lines were prepared by differential centrifugation using previously described protocols [45,47]. Briefly, cell homogenates were prepared by resuspending pelleted cells in PBS, followed by five rounds of freeze–thaw cycles prior to homogenization. Recombinant microsomes (rUGTs) were prepared by centrifuging the cell homogenate at a low speed (9000× *g* for 30 min) followed by ultracentrifugation (34,000× *g* for 60 min), with the total protein subsequently determined using a BCA assay. Recombinant microsomes (UGT1A9 and 2B7) and homogenates (UGT2B15) were used for inhibition studies.

### 2.3. Separation of R- and S-Oxazepam Glucuronides Using LC–MS/MS

Oxazepam and oxazepam glucuronides were identified using an ultra-pressure liquid chromatography system (UPLC; Waters Acquity; Waters Corp, Milford, MA, USA) coupled to a triple quadrupole mass spectrometer (MS; Waters Xevo TQD; Waters Corp) by multiple reaction monitoring (MRM). A Waters Corp BEH C18 Acquity UPLC column (1.7 µM, 2.1 × 100 mm) was used at a constant temperature of 40 °C for chromatographic separation. An elution gradient was used for 6.5 min with mobile phases A (5 mM ammonium formate) and B (100% methanol) under the following conditions: 90% A for 0.5 min, followed by a linear gradient for 3 min to 60% A, 1 min at 5% A, and re-equilibration for 1 min at 90% A.

The mobile phase was delivered at a flow rate of 0.4 mL/min, and the sample injection volume was 5 µL. The mass spectrometric analysis was performed via electrospray ionization operating in positive-ion mode with the capillary voltage at 0.6 kV, and the mass transitions of mass/charge ratios of 286.9 > 240.9 and 463.3 > 269.1 were used for oxazepam and oxazepam glucuronides, respectively. Ultrapure argon was used for collision-induced dissociation. The desolvation temperature was 500 °C, with 600 L/h of nitrogen gas for desolvation and 30 L/h for the cone, while the temperature of the source was 120 °C. Analytes were detected with dwell times of 0.063 s, and the collision energies and cone voltages were 22 V and 27 V for oxazepam and 25 V and 25 V for oxazepam glucuronides, respectively. 

### 2.4. In Vitro Inhibition Assays of R- and S-Oxazepam Glucuronidation

A preliminary screening assay was conducted to identify the cannabinoids and cannabinoid metabolites that inhibited R- and S-oxazepam glucuronidation at two concentrations of the cannabinoid: 10 μM and 100 μM. The assay mix included microsomes or homogenate from a UGT-overexpressing HEK293 cell line (120–200 µg), HLM or HKM (20 µg protein), substrate (R,S-oxazepam), 50 mM Tris-HCL buffer (pH 7.4), 5 mM MgCl_2_, 2% BSA, and 4 mM UDPGA in a final reaction volume of 30 µL. The microsomes were preincubated with alamethicin (50 µg/mg of microsomal protein) for 20 min on ice prior to the addition of the reaction components to permeabilize the microsomal membranes, since UGTs are located on the luminal side of endoplasmic reticulum. The oxazepam concentrations were below or close to the Km values reported for the corresponding enzyme source: 12 μM for both HLM and HKM, 60 μM for rUGT2B7 microsomes, 4 μM for rUGT1A9 microsomes, and 12 μM for rUGT2B15 homogenate [2]. The reaction was initiated by the addition of 4 mM UDPGA as the cofactor and incubated for 2 h at 37 °C. The reaction was terminated by adding 30 µL of an ice-cold stop solution (acetonitrile/methanol; 1:1). The reaction mixtures were centrifuged at 17,000× *g* for 30 min at 4 °C, and the supernatant was transferred to UPLC sample glass vial for analysis using UPLC–MS/MS (described above). As cannabinoids exhibit extensive nonspecific binding (70–90%) to proteins and labware, low-binding, 0.6 mL microcentrifuge tubes were used for all reactions. As a positive control for inhibition assays, ketoconazole (10 μM and 100 µM) was used as a potential inhibitor of the UGTs 1A9, 2B7, and 2B15. Reactions containing only the cannabinoid vehicle (3% methanol) and without any inhibitor (cannabinoids) were used as a baseline reaction for 100% enzyme activity, while a reaction without UDPGA was used as a negative control. All experiments were performed in three independent experiments. Prior to performing the inhibition studies, the microsomal protein amounts used and the incubation times were optimized for HLM, HKM, and recombinant microsomes/homogenates based on the following criteria: (1) R- and S-oxazepam glucuronide formation was linear with time and protein concentration; (2) oxazepam depletion was less than 20% of the initial amount; and (3) the UPLC–MS/MS method used to detect R,S-oxazepam glucuronide was robust and reproducible in detecting metabolite formation.

### 2.5. Identification of R,S-Oxazepam Glucuronides by β-Glucuronidase Hydrolysis

The R- and S-oxazepam glucuronides were confirmed with differential enzymatic hydrolysis. Compared to its S-diastereoisomers, benzodiazepine R-glucuronide diastereoisomers are more resistant to hydrolysis by β-glucuronidase from *E. coli* [5,48]. The reaction conditions were the same as mentioned above. Briefly, R,S-oxazepam was incubated with UDPGA and HLM and terminated with an ice-cold stop solution (acetonitrile/methanol; 1:1). Following centrifugation, 10 µL of the supernatant was dried down with a SpeedVac, and the identities of oxazepam glucuronide diastereomers were confirmed by their differential sensitivity to β-glucuronidase by incubating 1 µL of *E. coli* β-glucuronidase (7 units/mL) in a reconstituted 10 µL reaction (with water) at 37 °C for 10 min. A control reaction was conducted in the same manner, except that water was added instead of β-glucuronidase. Following centrifugation, 5 µL of supernatant was injected into the UPLC column for the analysis of the remaining individual oxazepam glucuronide diastereomers based on the peak area.

*Determination of IC_50_ and K*_i_ *Values*. For cannabinoids or metabolites that inhibited enzyme activity by ≥50% at either of the two cannabinoid concentrations used in the initial screenings, IC_50_ determinations were performed in HLM, HKM, and recombinant microsomes using multiple concentrations of cannabinoids ranging from 0.1–200 µM. All analyses were performed in three to six independent experiments. The nonspecific binding constants (f_u,inc_) for cannabinoids in HEK293 microsomes, HLM, and HKM were previously determined [40,41] and used to calculate the inhibition by unbound (‘free’) cannabinoid in each reaction. A nonlinear regression analysis and the following equation were used to calculate the IC_50_ values:

(1) $$y\;=\;Bottom\;+\left(Top\;-\;Bottom\right)\;/\;\left(1\;+\;10^{\left(x\;-\;LogIC_{50}\right)}\right)A$$

where ‘Bottom’ is the maximum observed inhibition (lowest percent activity) and ‘Top’ is the minimum observed inhibition (highest percent activity) for a given cannabinoid.

The inhibition constant (K_i_) was generated from the in vitro IC_50_ values assuming competitive inhibition (based on the Cheng–Prusoff equation; [49]). Considering that there have been no reports of the irreversible inhibition (e.g., mechanism-based inhibition) of UGTs, competitive inhibition was assumed as the worst-case scenario (highest inhibition) among all feasible types of reversible inhibitions [50,51]. The substrate concentration used in the incubations was below the reported K_m_; therefore, the K_i_ values were predicted using Equation (2):

(2) $$K_{i}=\frac{{IC}_{50}}{\frac{S}{K_{m}}+1}$$

where K_m_ is Michaelis–Menten constant for the substrate, S is the substrate concentration, and IC_50_ is half-maximal inhibitory concentration.

R- and S-oxazepam glucuronide formation was determined using TargetLynx software (Version 4.1, Waters Acquity) and further analyzed using Excel (Microsoft, Redmond, WA, USA). For each inhibitor (cannabinoid) concentration used, the amount of R- and S-oxazepam glucuronide formed was calculated relative to the control as the % relative activity (% metabolite formation) using the following formula:

% Relative activity = (peak area of R- and S-oxazepam glucuronide with inhibitor/

peak area of R- and S-oxazepam glucuronide without inhibitor) × 100%

The % of R- or S-oxazepam glucuronide formation vs. the log concentration of cannabinoid (inhibitor) was plotted to determine the IC_50_ values using GraphPad Prism 7.04 software (GraphPad Software Inc., San Diego, CA, USA).

An IC_50_ analysis was performed utilizing top and bottom constraints (top = 100 and bottom = greater than 0). Since cannabinoids bind to plastic and proteins, all IC_50_ and K_i_ values were corrected for nonspecific binding to determine the binding-corrected inhibition potency, also known as IC_50,u_ and K_i,u_, using the following equations:

IC_50,u_ = IC_50_ × f_u,inc._ (3)

K_i,u_ = f_u,inc_ × K_i_ (4)

### 2.6. Prediction of Potential In Vivo DDIs Based on In Vitro–In Vivo Extrapolation (IVIVE)

The potential in vivo effects of cannabinoids and their metabolites on R- and S-oxazepam metabolism were evaluated by using a mechanistic static model of reversible inhibition in accordance with the FDA guidelines [52]. The predicted magnitude of the increase in R- and S-oxazepam exposure was calculated as the ratio of the areas under the plasma concentration–time curves (AUCRs) with (AUC_i_) and without (AUC_0_) cannabinoid coadministration. The AUCR (magnitude of DDI) is predicted using Equation (5) [52]:

(5) $$AUCR=\frac{{AUC}_{i}}{{AUC}_{0}}=\left(\frac{1}{A_{h}\times f_{m}+\left(1-f_{m}\right)}\right)$$

with

(6) $$A_{h}=\frac{1}{1+\frac{{\left[I\right]}_{h}}{K_{i,u}}}$$

where A_h_ is the effect of reversible inhibition in the liver (Equation (6) [52], f_m_ is the fractional metabolism of R- and S-oxazepam via glucuronidation [1], and K_i,u_ is the estimated inhibition constant (see above). The f_m_ of R,S-oxazepam was set at 0.8 [53]. The maximum unbound plasma concentration at the inlet to the liver (I_h_) was calculated using Equation (7) [52,54]:

(7) $$\left[I_{h}\right]=f_{u,p}\times \left(C_{max}+\frac{F_{a}\times F_{g}\times K_{a}\times Dose}{Q_{h}\times R_{b}}\right)$$

The unbound concentration of the drug in plasma denoted by f_u,p_ [52] was set at 0.03 for THC [41,55], 11-OH-THC, and CBD [56]. The maximal concentration of the drug in plasma, including both free and bound, is denoted as C_max_ (described below) [52]. F_a_ represents the proportion of the dose absorbed from the intestinal lumen to the dose absorbed from the gut [52], and K_a_ indicates the absorption rate constant [57], which was assumed to be 1 and 0.02, respectively [52].

R_b_ (set to 0.4) is the ratio between the drug concentration in the blood and the drug concentration in the plasma [41,58], and Q_hep_ is the hepatic blood flow = 1500 mL/min [41,52]. The doses and the C_max_ values for THC, 11-OH-THC, and CBD used were the same as previously reported [20,57,59]. Briefly, to calculate the AUCR, we used three different average doses (low, high, and maximum) for two different routes of administration (inhalation and oral) [20,57,59]. The oral doses for CBD were 70 mg, 700 mg, and 2000 mg, and were 20 mg, 130 mg, and 160 mg for THC and 11-OH-THC [20]. The inhaled doses of CBD were 2 and 19 mg [57], whereas the doses for THC and 11-OH-THC were 25 mg, 70 mg, and 100 mg [20]. An AUCR value ≥ 1.25 was used according to the FDA guidance as an indicator of a strong potential for an in vivo DDI [52].

## 3. Results

*Identification of the R- and S-oxazepam glucuronides.* The glucuronide peaks were identified by the differential effect of enzymatic hydrolysis by β-glucuronidase and detected using UPLC–MS/MS (Figure 2). The peak with a retention time (RT) of 3.23 min was more resistant to β-glucuronidase, whereas the peak at RT 3.49 min was more sensitive to hydrolysis (Figure 2). Given that previous studies suggested that the S-oxazepam glucuronide was more sensitive to β-glucuronidase treatment [48], the two peaks were identified as R-oxazepam glucuronide (3.23 min RT) and S-oxazepam glucuronide (3.49 min RT), respectively. Additionally, it was reported that when separated by reverse-phase HPLC, the R-glucuronides of benzodiazepines typically elute prior to the S-glucuronides [5,48]. The identities of the oxazepam glucuronides and enzymes responsible for catalyzing the formation of the two glucuronide metabolites were further validated by incubating R,S-oxazepam with microsomes from the rUGTs 1A9, 2B7, and 2B15 (Figure 2). The incubation with rUGT1A9 microsomes resulted in the formation of primarily R-oxazepam glucuronide (3.22 min RT), while S-oxazepam glucuronide was the primary glucuronide formed with rUGT2B15 microsomes (3.48 min RT). In contrast, both R- and S-oxazepam glucuronides were formed during the incubation with rUGT2B7 microsomes. These results are consistent with previous studies indicating that while the UGTs 1A9 and 2B15 are stereoselective for oxazepam isomers, UGT2B7 is involved in the glucuronidation of both the R- and the S-oxazepam isomers [2].

**Figure 3:**
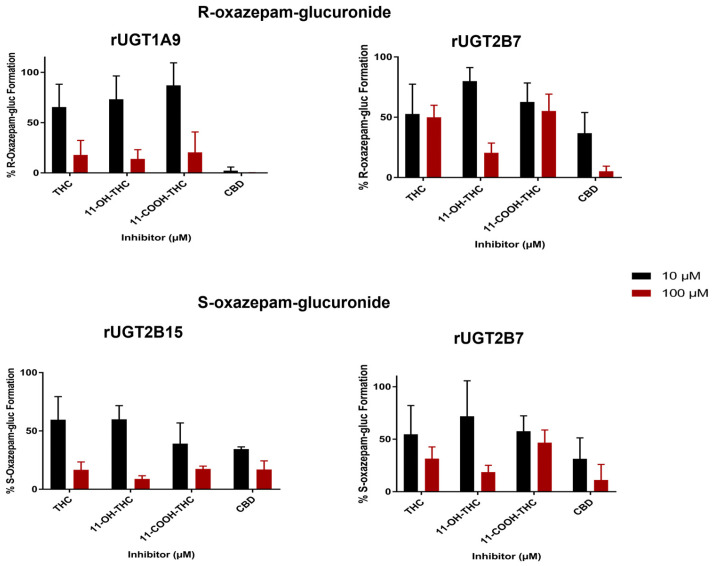
Inhibition screening of R- and S-oxazepam glucuronide formation by major cannabinoids and metabolites in rUGT1A9, rUGT2B15, and rUGT2B7 microsomes. Incubations were performed using 10 or 100 µM of cannabinoid/metabolite, with oxazepam concentrations at or below the known K_m_ value for the corresponding enzyme system. Each bar represents the mean ± standard deviation of triplicate independent assays. Results are expressed as a percentage of metabolite formed in incubations with cannabinoid compared with incubations without cannabinoid.

*Inhibition of R- and S-oxazepam glucuronidation.* The inhibitory effects of major cannabinoids (THC and CBD) and THC metabolites (11-OH-THC and 11-COOH-THC) on R- and S-oxazepam metabolism using rUGTs are shown in Figure 3. The preliminary screening results showed that at 100 µM THC, 11-OH-THC, or 11-COOH-THC, R-oxazepam glucuronide formation was inhibited in rUGT1A9 microsomes by 83%, 87%, and 80%, respectively, as compared to the control incubations without the addition of cannabinoids. CBD was the most potent inhibitor of R-oxazepam glucuronide formation in rUGT1A9 microsomes, with 10 and 100 µM CBD resulting in 98% and 99% decreases in R-oxazepam glucuronide formation, respectively, as compared to the incubations without CBD.

A similar inhibition pattern was observed with S-oxazepam glucuronide formation in rUGT2B15 microsomes for THC, 11-OH-THC, 11-COOH-THC, or CBD, with 10 µM and 100 µM cannabinoid exhibiting between 40 and 66% and 83 and 91% inhibition of S-oxazepam glucuronide formation, respectively, as compared with the control reactions (Figure 3). Additionally, a strong inhibition of both R- and S-oxazepam glucuronide formation was observed when 10 and 100 µM cannabinoids were used as inhibitors in incubations with rUGT2B7 microsomes, with 100 µM THC, 11-OH-THC, 11-COOH-THC, or CBD inhibiting R-oxazepam glucuronidation by 50%, 80%, 45%, and 95%, respectively, and S-oxazepam glucuronidation by 69%, 81%, 55%, and 89%, respectively. As a control for the inhibition assays, 100 µM ketoconazole was used as an inhibitor in separate assays containing microsomes from the rUGTs 1A9, 2B7, and 2B15, inhibiting oxazepam glucuronide formation by 49–85% in microsomes for the three enzymes.

To further investigate the inhibition potential of cannabinoids on oxazepam metabolism, the IC_50_ values were established in rUGT microsomes for THC, CBD, and 11-OH-THC and corrected for unbound cannabinoids using the nonspecific binding constants (f_u,inc_) identified for each cannabinoid in previous studies [40,41], as described in the Materials and Methods. The IC_50,u_ values for microsomes from the rUGTs 1A9, 2B7, and 2B15 were further validated using commercially prepared, pooled HLM and HKM (Table 1). The unbound fractions (f_u,inc_) for THC, CBD, and 11-OH-THC in overexpressing HEK293 cell lines reported were 0.042 ± 0.003, 0.038 ± 0.002, and 0.078 ± 0.042, respectively [40,41]. The f_u,inc_ of 11-OH-THC, CBD, and THC in the incubation mixtures with HKM were 0.094 ± 0.014, 0.062 ± 0.009, and 0.052 ± 0.005, respectively, and for HLM, the unbound fractions were 0.094 ± 0.014 (identical to that observed in HKM), 0.051 ± 0.008, and 0.048 ± 0.002, respectively [40,41].

**Figure 4:**
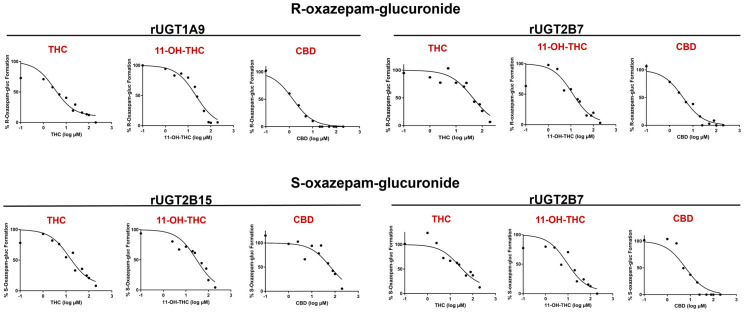
Representative IC_50_ curves for the inhibition of R- and S-oxazepam glucuronide formation by THC, 11-OH-THC, and CBD in rUGT2B7, rUGT1A9, and rUGT2B15 microsomes. R- and S-oxazepam glucuronide formation was measured using multiple concentrations of cannabinoids ranging from 0.1–200 µM. Transformed log (µM) concentrations of cannabinoids or metabolites are plotted against the % of R- and S-oxazepam glucuronide formation.

Representative IC_50_ curves of all cannabinoids tested in rUGT 1A9, 2B7, and 2B15 microsomes are shown in Figure 4, with the curves for HLM and HKM shown in Supplementary Figure S1. CBD demonstrated a strong inhibition of R-oxazepam glucuronidation in rUGT1A9 and rUGT2B7 microsomes, with IC_50,u_ values of 0.053 ± 0.011 μM and 0.10 ± 0.045 μM, respectively (Table 1). Additionally, the inhibitory effect of CBD on R-oxazepam glucuronidation was further validated in HLM and HKM, resulting in similarly low IC_50,u_ values of 0.84 ± 0.39 μM and 0.36 ± 0.062 μM, respectively (Table 1). For the inhibition of S-oxazepam glucuronidation, lower IC_50,u_ values of 0.11 ± 0.11 μM and 0.98 ± 0.48 μM were observed for rUGT2B7 microsomes and HKM, respectively, as compared to those observed for rUGT2B15 microsomes (2.0 ± 0.35 μM) and HLM (4.5 ± 2.4 μM; Table 1).

The major active THC metabolite, 11-OH-THC, demonstrated a similar inhibitory pattern to that of CBD. 11-OH-THC similarly showed a strong inhibition of R-oxazepam glucuronide formation in rUGT1A9 and 2B7, with IC_50,u_ values of 1.0 ± 0.60 μM and 0.77 ± 0.64 μM, respectively, with comparable IC_50,u_ values of 1.7 ± 0.47 μM and 1.7 ± 0.40 μM observed for the incubations containing HLM or HKM, respectively (Table 1). Similar to those observed for CBD, the IC_50,u_ values for the inhibition of S-oxazepam glucuronidation by 11-OH-THC in rUGT2B7 microsomes and HKM of 0.53 ± 0.26 μM and 1.8 ± 0.19 μM, respectively, were lower than those observed for rUGT2B15 microsomes and HLM (IC_50,u_ = 4.5 ± 2.1 μM and 3.9 ± 2.4 μM, respectively; Table 1). 

As shown in Table 1, THC demonstrated a strong inhibition of R-oxazepam glucuronidation in rUGT 1A9 and 2B7 microsomes as well as in HLM and HKM, with IC_50,u_ values of 0.50 ± 0.31 μM, 1.4 ± 0.67 μM, 1.6 ± 0.77 μM, and 4.5 ± 1.2 μM, respectively. While weak inhibition (IC_50_ > 100 μM) of S-oxazepam glucuronide formation was observed for THC in HLM and HKM, THC exhibited a relatively strong inhibition of S-oxazepam glucuronidation in rUGT2B7 (IC_50,u_ = 1.4 ± 0.28 μM) and rUGT2B15 (IC_50,u_ = 0.84 ± 0.42 μM) microsomes (Table 1). The relative levels of inhibition observed by CBD, 11-OH-THC, and THC on R- vs. S-oxazepam glucuronide formation as determined by IC_50,u_ value determinations in rUGT2B7 microsomes were similar.

The K_i_ values for THC, CBD, and 11-OH-THC were estimated using the IC_50_ values generated in vitro in HLM (described above) and assumed competitive inhibition (Table 2). Similar to calculated IC_50_ values, K_i_ values were corrected for nonspecific binding (K_i,u_) using the f_u,inc_ values described above [40,41]. 11-OH-THC, CBD, and THC strongly inhibited R-oxazepam glucuronide formation in HLM, with estimated K_i,u_ values ranging from 0.82 to 1.7 μM (Table 2); K_i,u_ values for S-oxazepam were slightly higher (>3.2 μM).

*Prediction of potential clinical drug–drug interactions using mechanistic static modeling.* The area under the concentration–time curve ratio (AUCR) was calculated utilizing the estimated K_i.u_ to predict a potential DDI between oxazepam and major cannabis constituents. The K_i,u_ values for S-oxazepam glucuronide formation were utilized, since it is considered to be the major metabolite in vivo [2,8]; however, the K_i,u_ values for R-oxazepam glucuronide were also used to predict ‘worst-case’ DDI scenarios (i.e., maximum inhibition). A graphical representation of predicted AUCRs for R- and S-oxazepam are shown for oral and inhaled CBD, 11-OH-THC, and THC in Supplementary Figure S2, with the K_i,u_ values of both R- and S-oxazepam glucuronide formation (see Table 2) taken into consideration. The cannabinoid doses correspond to the CBD or THC doses specified in previous clinical trials, case reports, and on vendor websites [20], and the cannabinoid concentrations and doses used to predict the AUCRs of oxazepam in the presence vs. absence of cannabinoids are shown in Table 3. Using K_i,u_ values for the inhibition of S-oxazepam glucuronidation in the mechanistic static model, oral CBD at high (700 mg) and maximum (2000 mg) doses [20] resulted in a DDI with oxazepam, with AUCRs of 1.44 and 2.03, respectively; no DDI (AUCR ≤ 1.25) was predicted with oxazepam at a low oral CBD dose (70 mg) [20]. No in vivo DDIs were predicted for the low and high inhaled CBD doses [57] used in previous trials. 11-OH-THC exhibited no DDI (AUCR ≤ 1.25) with S-oxazepam at low, high, and maximum oral and inhaled THC doses. 

Using K_i,u_ values for the inhibition of R-oxazepam glucuronidation in the mechanistic static model, oral CBD at low doses (70 mg) resulted in no predicted DDI with oxazepam; a potential for a clinically relevant DDI with oxazepam was predicted for oral CBD at high (700 mg) and maximum (2000 mg) doses, with AUCRs of 2.42 and 3.45, respectively [20]. After the oral administration of THC at a maximum dose of 160 mg, the predicted AUCR was 1.25. All other oral and inhaled doses of THC showed no potential for a clinically relevant DDI for both THC and 11-OH-THC with oxazepam, with the AUCR ≤ 1.25. Despite not meeting the AUCR threshold of 1.25, higher doses of THC led to an increase in the AUCRs for both THC and 11-OH-THC when administered alongside oxazepam.

## 4. Discussion

Three UGT enzymes were previously reported to be responsible for oxazepam glucuronidation, with UGTs 1A9 and 2B7 active in R-oxazepam glucuronide formation and UGTs 2B15 and 2B7 active in S-oxazepam glucuronide formation [2]. In the present study, a strong inhibitory effect of major cannabinoids on the UGT-mediated glucuronidation of R,S-oxazepam metabolism was demonstrated. Of the cannabinoids tested, CBD exhibited the highest level of inhibition of the UGT1A9- and UGT2B7-mediated formation of R-oxazepam glucuronide, with both THC and 11-OH-THC exhibiting more moderate levels of inhibition. Interestingly, THC exhibited the highest level of inhibition towards UGT2B15-mediated S-oxazepam glucuronide formation but was less inhibitory than CBD and 11-OH-THC in inhibiting UGT2B7-mediated S-oxazepam glucuronide formation.

Consistent with the patterns of inhibition observed for two THC metabolites tested in previous studies using probe inhibitors [40], marginal or no inhibition (IC_50_ > 100 µM) was observed by 11-COOH-THC in the recombinant UGT microsomes, HLM, or HKM tested in the present study. However, in contrast to the lack of inhibitory activity observed previously for 11-OH-THC against UGT microsomes using probe substrates [40], 11-OH-THC exhibited an inhibition of rUGT1A9, 2B7, and 2B15 as well as HLM and HKM when R,S-oxazepam was used as substrate. Previous studies by Anderson et al., 2021 [60] reported CYP450 inhibition to be highly substrate dependent, and this may also be the case for some UGT enzymes.

The UGTs 2B7, 2B15, and 1A9 are well expressed in the liver, representing approximately 17%, 11%, and 8% of total hepatic UGT expression, respectively [61]. The similar IC_50,u_ values observed for THC and 11-OH-THC for R-oxazepam glucuronide formation in microsomes from rUGTs 2B7 and 1A9 vs. those observed in HLM are consistent with their relatively high levels of hepatic expression. While a strong inhibition of R-oxazepam glucuronide formation was observed by CBD in microsomes from the rUGTs 2B7 and 1A9 and HLM, the IC_50,u_ observed for HLM was 8–16-fold higher than that observed for the microsomes for either recombinant enzyme. The IC_50,u_ value that most closely matched that which was observed in HLM for R-oxazepam glucuronide formation was observed with UGT2B7, which is consistent with the higher level of hepatic expression of UGT2B7 as compared to UGT1A9.

By comparison, the IC_50,u_ values observed for HLM for S-oxazepam glucuronide formation were similar to those observed in rUGT2B15 microsomes, consistent with UGT2B15 being the most active enzyme in S-oxazepam glucuronide formation [2] and its relatively high level of hepatic expression. This was observed for both CBD and 11-OH-THC; as we were not able to determine accurate IC_50,u_ values for THC, such a comparison could not be performed for this cannabinoid.

The UGTs 1A9 and 2B7 are expressed at similar levels in the kidneys (45% and 41% of total renal UGT expression, respectively), with UGT2B15 exhibiting little to no kidney expression [62]. For R-oxazepam glucuronide formation, the IC_50,u_ values observed with CBD, 11-OH-THC, and THC in HKM were generally most similar to those observed in rUGT2B7 microsomes as compared to that observed in UGT1A9 microsomes. Given the low expression of UGT2B15 in the kidneys, it is therefore likely that the inhibition of S-oxazepam glucuronide formation observed by cannabinoids in HKM is due to inhibition of UGT2B7.

The estimated K_i,u_ values observed in HLM for S- and R-oxazepam glucuronidation by CBD, 11-OH-THC, and THC are in the micromolar range (0.82 to 3.7 µM), with the K_i,u_ values observed for S-oxazepam glucuronidation approximately 2- to 5-fold higher as compared to those observed for R-oxazepam glucuronidation.

When compared to the physiologically achievable cannabinoid plasma concentration corrected for plasma protein binding (C_max,u_), the K_i,u_ values suggest that a potential DDI may occur after the concomitant usage of a cannabinoid such as CBD with oxazepam. For instance, the K_i,u_ values for CBD approached the physiological levels of CBD in the plasma, corrected for plasma protein binding, after a single dose of CBD (C_max,u_ = 0.13 µM) [37]. Additionally, Taylor et al. (2018) [37] observed a C_max,_ of 4.4 µM for a 1500 mg dose of CBD following a high-fat diet.

In addition, the estimated K_i,u_ values for THC and 11-OH-THC in the present study were only slightly greater or close to the observed C_max,u_ values from previous studies [20]. The C_max_ of THC and 11-OH-THC were 0.64 µM and 0.09 µM after smoking and 0.53 µM and 0.16 µM following the oral ingestion of 50 mg THC, respectively [63]. Huestis et al. (1992) [64] found that smoking cannabis cigarettes with 3.55% THC resulted in a similar C_max_ value for THC (0.515 µM).

These data are consistent with the results obtained in the present study from mechanistic static modeling, which showed a potential for DDIs with these cannabinoids (CBD and THC) and oxazepam metabolism. However, with chronic or frequent dosing of cannabis, C_max,u_ is likely higher than those reported from clinical studies, increasing the likelihood of a stronger DDI. Other factors that should be considered when assessing the potential for DDIs of cannabinoids and interpreting the in vitro inhibition potency values (IC_50,u_ and K_i,u_) include the cannabis strain, dosage, frequency (frequent vs. occasional user), route of administration (inhaled vs. oral), and user expertise [19]. The plasma concentrations (C_max_) may vary between individuals, even within the same route of administration, depending on parameters such as the number and duration of puffs, the puff hold time, and the volume of inhalation [35,65,66]. It is also important to consider that cannabis products intended for medical use are standardized, whereas the nonmedical grade products are non-standardized and contain unregulated levels of THC or CBD. In addition, it is important to consider the impact of genetic variation when investigating DDIs. For instance, a common missense polymorphism in the UGT2B15 enzyme (D85Y) resulted in decreased oxazepam clearance [2,6], which could potentially lead to an increased risk of DDIs and adverse effects such as sedation. Therefore, the incorporation of genetic variation into DDI studies should be considered.

The mechanistic static modeling predicted a potential clinically significant interaction between CBD and oxazepam, with AUCR values ranging from 1.44 to 3.45 for high and maximum doses of CBD specified in previous clinical trials, case reports, and on vendor websites [20]. Due to its relatively low inhibitory activity for S-oxazepam glucuronide formation, THC (160 mg) was only predicted to cause a DDI with oxazepam when using its K_i,u_ value for R-oxazepam glucuronide formation. 11-OH-THC was not predicted to cause a significant DDI at oral and inhaled doses of THC.

A potential limitation of the present study is the high lipophilicity of cannabinoids and their high affinities for proteins and labware. This makes the determination of the extent of nonspecific binding of an individual cannabinoid in an experimental system challenging, potentially leading to the underestimation of kinetic parameters such as IC_50_ or K_i_ values. However, the predictions described in the present study are consistent with the few studies that reported a clinical interaction between CBD and commonly used BZDs. For instance, oral CBD (Epidiolex) inhibited the CYP-mediated metabolism of clobazam, a well-known BZD utilized for its anticonvulsant properties. After oral administration of Epidiolex (5–25 mg/kg/day) and clobazam to patients with refractory epilepsy, the plasma concentration of the active clobazam metabolite, N-desmethylclobazam, increased significantly (approximately 5-fold) [44]. Similarly, Morrison et al. (2019) [67] reported that the coadministration of clobazam, stiripentol, and CBD (750 mg twice daily) resulted in a 3.4-fold increase in the N-desmethylclobazam concentration. Another study showed an interaction between oral CBD (750 mg twice daily) and the sedative benzodiazepine midazolam [43]. Midazolam is metabolized via CYP450s to 1-hydroxymidazolam, which is subsequently glucuronidated by UGT2B7 [68]; the concurrent administration of CBD and midazolam resulted in a 12% increase in the level of 1-hydroxymidazolam [43].

The potential limitations of the present study also include the fact that the spontaneous racemization of oxazepam has been reported in an aqueous solution at 37 °C with a half-life of less than 4 min [2,69], which is much shorter than the incubation times used in the present study (30–120 min). Therefore, purified stereoisomers of oxazepam (R- and S-) cannot be used for the studies outlined here (i.e., all oxazepam preparations will become racemic after a few minutes at 37 °C). BSA was also added to the incubation to increase the solubilities of the cannabinoids, the activities of recombinant enzymes, as well as to sequester inhibitory, long-chain, unsaturated fatty acids. While oxazepam has been reported to bind to BSA [2], we did not have any sensitivity issues in detecting metabolite formation in our reactions. In addition, the present study examined the inhibition potency of each cannabinoid independently, although a combination of cannabinoids in the incubation mixture could enhance the inhibition potency and would be more physiologically similar to someone who consumes cannabis. Qian et al. (2019) [39] reported such a pattern when THC, CBD, and CBN were combined as inhibitors of CES1 in vitro. Finally, it is important to acknowledge the limitations of mechanistic static modeling, which only examines a single inhibitor (i.e., cannabinoid) concentration, with the combined effects of the many different cannabinoids in cannabis not examined together. However, a physiologically based pharmacokinetic (PBPK) model would provide a more realistic estimate of the potential DDI by considering dynamic variations in the inhibitor (e.g., cannabinoid) concentration over time, as well as the combined effects of multiple cannabinoids such as CBD and its metabolites.

Taken together, the in vitro and mechanistic static modeling data from the present study indicate a potential pharmacokinetic DDI when major cannabinoids like CBD or THC and oxazepam are administered concurrently. This may result in increased levels of oxazepam resulting in potentially significant side effects such as prolonged sedation. While additional in vitro–in vivo extrapolations (dynamic model) and clinical studies are needed to determine the clinical implications of these in vitro and computational findings and provide a more thorough evaluation of the DDI risk, the present studies can serve as early preliminary data for future clinical studies, which may aid clinicians in optimizing therapeutic regimens and patient care decisions.

## Figures and Tables

**Table 1:** IC_50_ and IC_50_,_u_ values (µM) of 11-OH-THC, CBD, and THC for inhibition of UGT-mediated metabolism of R,S-oxazepam.

		R-Oxazepam Glucuronide		S-Oxazepam Glucuronide	
Cannabinoid	Enzyme ^a^	IC_50_	IC_50,u_^b^	IC_50_	IC_50,u_^b^
	rUGT1A9	13 ± 7.7	1.0 ± 0.60	-	-
11-OH-THC	rUGT2B7	9.9 ± 8.2	0.77 ± 0.64	6.8 ± 3.3	0.53 ± 0.26
	rUGT2B15	- ^c^	-	58 ± 27	4.5 ± 2.1
	HKM	18 ± 4.3	1.7 ± 0.40	19 ± 2.0	1.8 ± 0.19
	HLM	18 ± 5.0	1.7 ± 0.47	41 ± 25	3.9 ± 2.4
	rUGT1A9	1.4 ± 0.29	0.053 ± 0.011	-	-
CBD	rUGT2B7	2.6 ± 1.2	0.10 ± 0.045	2.9 ± 2.9	0.11 ± 0.11
	rUGT2B15	-	-	54 ± 9.0	2.0 ± 0.35
	HKM	5.9 ± 1.0	0.36 ± 0.062	16 ± 7.7	0.98 ± 0.48
	HLM	16 ± 7.7	0.84 ± 0.39	87 ± 46	4.5 ± 2.4
	rUGT1A9	11 ± 7.5	0.50 ± 0.31	-	-
THC	rUGT2B7	33 ± 16	1.4 ± 0.67	33 ± 6.6	1.4 ± 0.28
	rUGT2B15	-	-	20 ± 10	0.84 ± 0.42
	HKM	94 ± 26	4.5 ± 1.2	>100	>4.8 ^d^
	HLM	34 ± 16	1.6 ± 0.77	>100	>5.2 ^d^

^a^ r, recombinant enzyme from overexpressing cell line microsomes; HKM, human kidney microsomes; HLM, human liver microsomes. ^b^ IC_50_,_u._ values are the means (± S.D.) in µM and are the IC_50_ values after correction with previous fraction unbound (f_u,inc_) values determined by Nasrin et al., 2021 [40,41] for the different cannabinoids tested. ^c^ Dashes indicate that values were not determined, because little or no corresponding oxazepam glucuronide isomer was formed by that corresponding UGT enzyme. ^d^ The f_u,inc_ for THC of 0.052 and 0.048 were used for calculating the minimum IC_50,u_ in HKM and HLM, respectively.

**Table 2:** K_i_ and K_i,u_ values (µM) of 11-OH-THC, CBD, and THC for inhibition of UGT-mediated metabolism of R,S-oxazepam.

		R-Oxazepam Glucuronide		S-Oxazepam Glucuronide	
Cannabinoid	Enzyme	K_i_^b^	K_i,u_^c^	K_i_^b^	K_i,u_^c^
11-OH-THC	HLM ^a^	18 ± 4.9 ^d^	1.7 ± 0.46	34 ± 21	3.2 ± 2.0
CBD	HLM	16 ± 7.4	0.82 ± 0.38	72 ± 32	3.7 ± 1.6
THC	HLM	33 ± 15	1.6 ± 0.74	>83 ^e^	>3.5 ^e^

^a^ HLM, human liver microsomes. ^b^ K_i_ values were generated from IC_50_ values assuming competitive inhibition. Substrate concentrations were at or below the reported K_m_ value from Court et al., 2002 [2]. The equation used was K_i_ = IC_50_/S/K_m_ + 1, where S is the substrate concentration. ^c^ K_i_,_u_ values were the K_i_ values after correction with previous fraction unbound (f_u,inc_) values determined by Nasrin et al., 2021 [40,41]. ^d^ Data represent the mean (±S.D.). ^e^ The f_u,inc_ for THC of 0.042 was used for calculating the minimum K_i,u_.

**Table 3:** Prediction of clinical UGT-mediated cannabis–oxazepam interaction using a mechanistic static model after oral or inhaled doses of THC or CBD.

Cannabinoid	Dose (mg) ^a^	Route of Administration	C_max_ (μM) ^a^	Predicted AUCR	
				R-Oxazepam ^c^	S-Oxazepam ^d^
CBD	70	Oral	0.089	1.21	1.05
	700	Oral	0.89	**2.42** ^e^	**1.44**
	2000	Oral	2.5	**3.45**	**2.03**
	2 ^b^	Inhalation	0.0064 ^b^	1.00	1.00
	19 ^b^	Inhalation	0.35 ^b^	1.07	1.02
THC	20	Oral	0.030	1.03	- ^f^
	130	Oral	0.20	1.20	-
	160	Oral	0.24	**1.25**	-
	25	Inhalation	0.25	1.04	-
	70	Inhalation	0.70	1.12	-
	100	Inhalation	0.99	1.17	-
11-OH-THC	20	Oral	0.015	1.03	1.02
	130	Oral	0.10	1.18	1.10
	160	Oral	0.12	1.22	1.12
	25	Inhalation	0.012	1.04	1.01
	70	Inhalation	0.032	1.10	1.05
	100	Inhalation	0.046	1.14	1.08

^a^ Doses and C_max_ values used to predict AUCR were reported from Bansal et al., 2022 [20]. Doses used for modeling 11-OH-THC were the same for administered doses of THC. ^b^ Doses and C_max_ used to predict AUCRs were reported from Cox et al., [57]. ^c^ K_i,u_ values for inhibition of R-oxazepam glucuronide formation were used to predict oxazepam AUCR. ^d^ K_i,u_ values for inhibition of S-oxazepam glucuronide formation were used to predict oxazepam AUCR. ^e^ Bold values represent the AUCR cutoff recommended by the FDA ≥1.25. ^f^ Dashes indicate that values were not determined because IC_50_ > 100 μM.

## Data Availability

The data presented in this study are available in this article (and in the Supplementary Materials).

## Supplementary Materials

The following supporting information can be downloaded at: https://www.mdpi.com/article/10.3390/pharmaceutics16020243/s1, Figure S1: Representative IC_50_ curves for the inhibition of R- and S-oxazepam glucuronide formation by THC, 11-OH-THC, and CBD in HLM and HKM. R- and S-oxazepam glucuronide formation was measured using multiple concentrations of cannabinoids ranging from 0.1–200 µM. Transformed log (µM) concentration of cannabinoids or metabolites are plotted against the % of R- and S-oxazepam glucuronide formation. As THC inhibited S-oxazepam glucuronide formation with an IC_50_ > 100, representative curves are not shown. Figure S2: Graphical representation of predicted magnitudes of increase in R- (blue) and S-oxazepam (orange) exposure with and without cannabinoid coadministration. AUCRs were calculated based on oral and inhaled doses (low, average, and high) of CBD and THC [20,57]. The dotted line represents the AUCR ≥ 1.25 cut-off recommended by the FDA for a potential DDI [52].
