# Supplementary material for: Cannabinoid-Induced Stereoselective Inhibition of R-S-Oxazepam Glucuronidation: Cannabinoid–Oxazepam Drug Interactions
Source: Pharmaceutics. 2024 Feb 7;16(2):243. doi: 10.3390/pharmaceutics16020243 (PMC10892093; doi:10.3390/pharmaceutics16020243)
Supplement: Supplementary file 1 [file pharmaceutics-16-00243-s001.zip › Supplementary Figure S1.pdf]

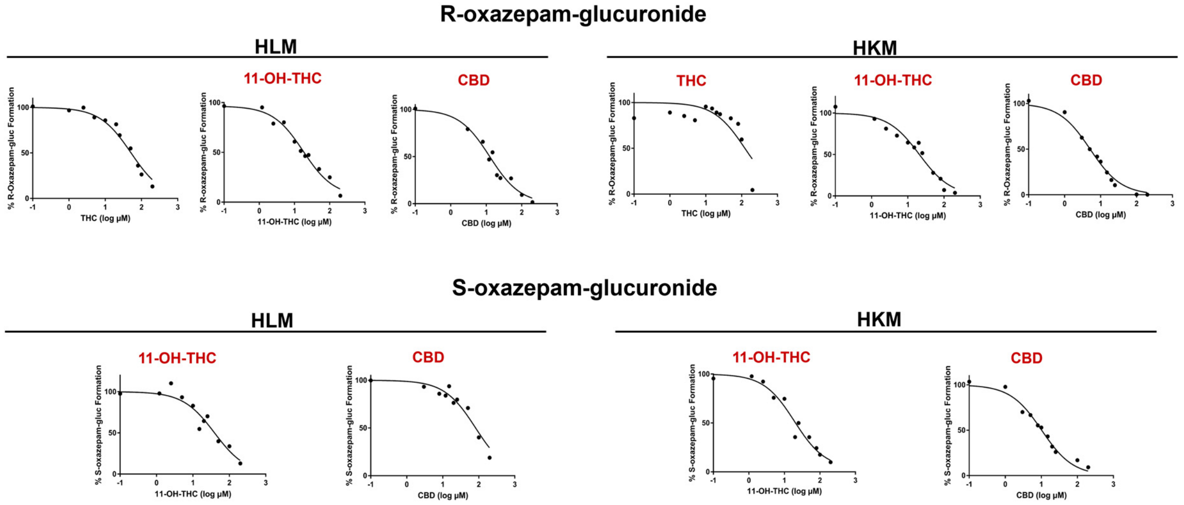


**Figure S1.** Representative $IC_{50}$ curves for the inhibition of R- and S-oxazepam glucuronide formation by THC, 11-OH-THC, and CBD in HLM and HKM. R- and S-oxazepam glucuronide formation was measured using multiple concentrations of cannabinoids ranging from 0.1–200 µM. Transformed log (µM) concentration of cannabinoids or metabolites are plotted against the % of R- and S-oxazepam glucuronide formation. As THC inhibited S-oxazepam glucuronide formation with an $IC_{50}$ > 100, representative curves are not shown.
