# Supplementary material for: Cannabinoid-Induced Stereoselective Inhibition of R-S-Oxazepam Glucuronidation: Cannabinoid–Oxazepam Drug Interactions
Source: Pharmaceutics. 2024 Feb 7;16(2):243. doi: 10.3390/pharmaceutics16020243 (PMC10892093; doi:10.3390/pharmaceutics16020243)
Supplement: Supplementary file 1 [file pharmaceutics-16-00243-s001.zip › Supplementary Figure S2.pdf]

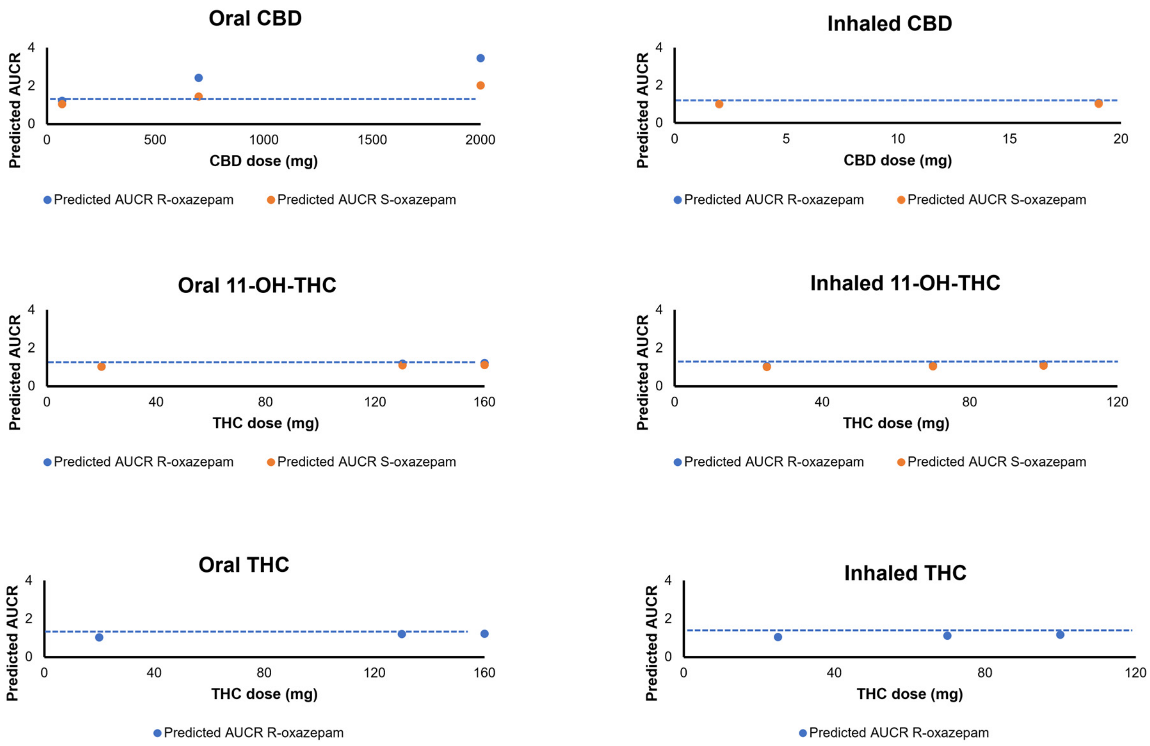

Figure S2: Graphical representation of predicted magnitudes of increase in R- (blue) and S-oxazepam (orange) exposure with and without cannabinoid coadministration. AUCRs were calculated based on oral and inhaled doses (low, average, and high) of CBD and THC [20,57]. The dotted line represents the AUCR ≥ 1.25 cut-off recommended by the FDA for a potential DDI [52].
